# Regression of Plaque Enhancement Within Symptomatic Middle Cerebral Artery Atherosclerosis: A High-Resolution MRI Study

**DOI:** 10.3389/fneur.2020.00755

**Published:** 2020-07-28

**Authors:** Wen-Jie Yang, Jill Abrigo, Yannie Oi-Yan Soo, Simon Wong, Ka-Sing Wong, Thomas Wai-Hong Leung, Winnie Chiu-Wing Chu, Xiang-Yan Chen

**Affiliations:** ^1^The Russell H. Morgan Department of Radiology and Radiological Sciences, The Johns Hopkins University, Baltimore, MD, United States; ^2^Department of Imaging and Interventional Radiology, Prince of Wales Hospital, The Chinese University of Hong Kong, Shatin, China; ^3^Department of Medicine and Therapeutics, The Chinese University of Hong Kong, Shatin, China; ^4^Department of Health Technology and Informatics, The Hong Kong Polytechnic University, Kowloon, China

**Keywords:** intracranial atherosclerosis, magnetic resonance imaging, stroke, inflammation, middle cerebral artery, plaque enhancement

## Abstract

**Objective:** Contrast enhancement is a vital feature of the intracranial atherosclerotic plaque on high-resolution magnetic resonance imaging (HRMRI), but its clinical significance is still unclear. We aimed to quantitatively assess plaque enhancement patterns in the middle cerebral artery (MCA) atherosclerotic plaque.

**Methods:** We conducted a cross-sectional study by prospectively recruiting stroke or transient ischemic attack patients with >30% of MCA stenosis of either side. All patients underwent contrast-enhanced HRMRI scans. Enrolled patients were classified into acute phase (<4 weeks), subacute phase (4–12 weeks) and chronic phase (>12 weeks) groups based on the time interval from stroke onset to imaging scan. Plaque enhancement index was calculated for each MCA lesion at the maximal narrowing site.

**Results:** We identified a total of 89 MCA plaques [53 (60%) symptomatic and 36 (40%) asymptomatic; 57 (64%) acute, 18 (20%) subacute and 14 (16%) chronic] in 58 patients on HRMRI. Among the acute lesions, symptomatic plaques had a significantly stronger plaque enhancement than asymptomatic plaques (symptomatic vs. asymptomatic: 38.9 ± 18.2 vs. 18.2 ± 16.2, *p* < 0.001). Among the symptomatic lesions, plaque enhancement diminished with increasing time after stroke onset (38.9 ± 18.2, 22.0 ± 22.8, and 5.0 ± 10.1 for acute, subacute, and chronic phase, respectively; *p* = 0.001).

**Conclusion:** Plaque enhancement in the acute atherosclerotic plaque is closely related to recent ischemic events. In symptomatic atherosclerosis, plaque enhancement regresses over time after ischemic stroke, which may offer the potential to monitor the plaque activity in intracranial atherosclerosis using HRMRI.

## Introduction

Intracranial atherosclerotic disease (ICAD) is a leading cause of ischemic stroke, especially among Asian stroke patients (1, 2). The challenge for current imaging modalities is not only to detect the presence of intracranial atherosclerotic plaque but also to clarify the high-risk lesions that are vulnerable to thrombosis. In addition to lumen stenosis that has long been used as an imaging standard for risk-stratifying patients, pathological studies reveal that some other plaque features, such as large lipid core, increased neovascularization, and plaque inflammation, also contribute to plaque instability and thromboembolic events (3, 4), highlighting the importance of visualizing the plaque components to predict future vascular events.

High-resolution magnetic resonance imaging (HRMRI) has gained prominence as an imaging approach to reliably visualize the vessel wall pathology and thus is an ideal imaging tool to identify plaque characteristics that are indicative of plaque instability (5, 6). Several clinical studies have been done to validate the utility of HRMRI in characterizing imaging features of high-risk intracranial atherosclerotic lesions, including plaque distribution, intraplaque hemorrhage (IPH), patterns of arterial remodeling (7–11). Contrast enhancement after intravenous gadolinium-based contrast administration can improve the accuracy of HRMRI in identifying the activity of atherosclerotic lesions by detecting inflammation and neovascularization (12, 13). Prior studies of extracranial atherosclerotic plaque have proposed plaque enhancement as an imaging indicator of plaque vulnerability and high stroke risk (14–16). Plaque enhancement has less been studied in ICAD. Some recent studies revealed the clinical significance of gadolinium enhancement of intracranial lesions by showing the independent association between plaque enhancement and symptomatic intracranial plaques (17–21). However, the time-course changes of contrast enhancement in intracranial atherosclerosis are still controversial. A few studies demonstrated that the strength of contrast enhancement in symptomatic plaques decreased with increasing time after ischemic stroke (22, 23), but the sample size is relatively small to draw a firm conclusion. In the present study, we aimed to investigate whether the strength of plaque enhancement in middle cerebral artery (MCA) atherosclerosis changes after the onset of ischemic stroke.

## Materials and Methods

### Study Population

The study was approved by the Joint Chinese University of Hong Kong-New Territories East Cluster Clinical Research Ethics Committee (The Joint CUHK-NTEC CREC). Consecutive patients who were admitted to the Prince of Wales Hospital from February 2014 to November 2016 were prospectively recruited. The inclusion criteria were as follows: (1) ischemic stroke in MCA territory confirmed by MRI or clinical evidence of transient ischemic attack (TIA) with ischemic symptoms corresponding to the vascular distribution of MCA; (2) >30% of MCA stenosis (in M1 or M2 segment) of either side as confirmed by MR angiography; (3) one or more atherosclerotic risk factors, including hypertension, diabetes mellitus, hyperlipidemia, and smoking. Patients with the following conditions were excluded: (1) contraindications to MRI; (2) non-atherosclerotic vasculopathy, such as arteritis, dissection, or Moyamoya disease; (3) evidence of >50% of extracranial stenosis diagnosed by digital subtraction angiography, MR angiography and/or Carotid Doppler; (4) evidence of cardioembolism, such as atrial fibrillation.

Patients were classified as acute patients if scans were performed within 4 weeks post the onset of symptoms, subacute if between 4 and 12 weeks from stroke onset, and chronic if it was performed beyond 12 weeks from the presentation. Acute and subacute patients were given mono antiplatelet treatment (aspirin 80 mg/day). Chronic patients were extracted from a previous study (24), in which patients were given dual antiplatelet (aspirin 80 mg/day and clopidogrel 75 mg/day) for a total of 4 weeks from the onset of stroke, followed by aspirin 80 mg/day alone. All patients received statin treatment (Atorvastatin, Simvastatin, or Rosuvastatin) with the targeting low-density lipoprotein of <70 mg/dL (1.8 mmol/L). Other drugs were used for risk factor management following the up-to-date guidelines (25).

### Imaging Protocol

MRI was performed using a 3T Achieva MR system (Philips Healthcare, Cleveland, OH, USA) with an 8-channel head coil. A transverse 3D T1-weighted (T1w) Volumetric ISotropically Turbo spin echo Acquisition (VISTA) sequence, before and after contrast administration, was obtained for all the patients. The following scan parameters were used for the T1w VISTA: field-of-view 200 × 167 × 45 mm^3^, acquired resolution 0.6 × 0.6 × 1.0 mm^3^, reconstructed resolution 0.5 × 0.5 × 0.5 mm^3^ using zero filling, repetition time (TR) 1,500 ms, echo time (TE) 36 ms, SENSE factor 1.5 (phase-encode direction), echo spacing 4.0 ms, TSE + startup echoes 56+6 and scan duration 6:51 min. The gadolinium-containing contrast agent (Dotarem, Gadoteric acid 0.5 mmol/mL; Guerbet, Roissy CdG Cedex, France) was administered to the patients (0.1 mL/kg), and contrast-enhanced T1w VIRTA sequence was performed ~5 min after injection was performed. A 3-dimensional Time-Of-Flight Magnetic Resonance Angiography (TOF-MRA) was obtained with the following parameters: FOV 200 × 200 × 56 mm^3^, acquired resolution 0.4 × 0.6 × 0.7 mm^3^, TR/TE 23/3.5 ms, and scan duration 3:07 min.

### Image Analysis

Two reviewers who were blinded to the clinical data analyzed the matched pre- and post-contrast T1-weighted images of MCA. The images were graded on a 3-point scale: 1 = poor, 2 = adequate, and 3 = excellent. Images with a score of 2 or 3 were analyzed after being zoomed to 400% by using VesselMass software (Leiden University Medical Center, The Netherlands).

The cross-sectional area of MCA plaques was measured at the site of maximal lumen narrowing on T1-weighted images. Vessel-cerebrospinal fluid interface was used to trace vessel area (VA) manually, and blood–intima interface was used to determine lumen area (LA). The wall area (WA) was estimated by subtracting LA from VA. Plaque load was calculated as WA/VA ^*^ 100%. LA of the lesion-free site at the MCA distal portion (LA _reference_) was measured as a reference to calculate the luminal stenosis as follows: luminal stenosis = (1 - LA _stenosis_/LA _reference_) ^*^ 100%. IPH was defined as an area of high signal (>150% of the adjacent area of the vessel wall) in the plaque, as described previously (10).

**Figure 1:**
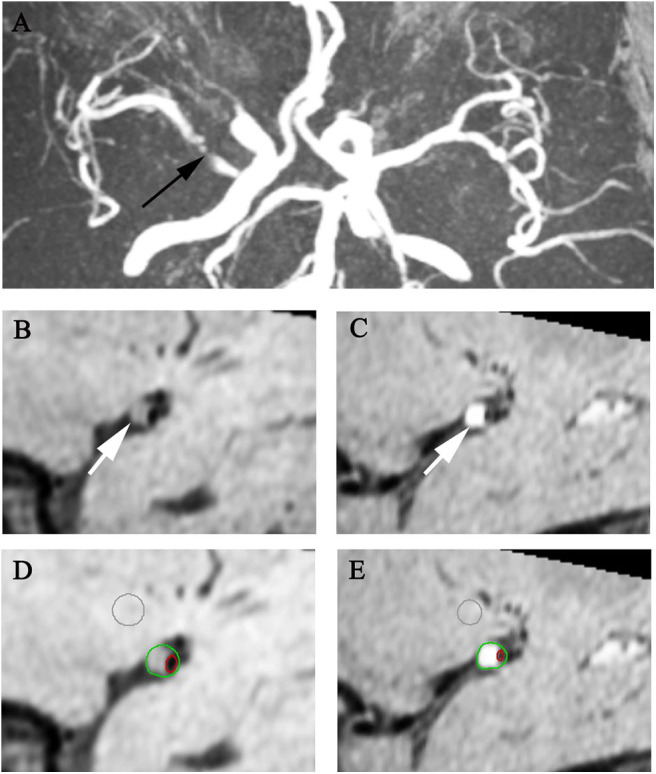
Representative MR images showing enhancement index measurement. A 60-year-old man with acute stroke showed severe right MCA stenosis on MRA (**A** arrow). After matching pre- **(B,D)** and post-contrast T1w images **(C,E)** at the most stenotic site, the plaque enhancement index of the plaque (arrows) is 48.74%.

**Figure 2:**
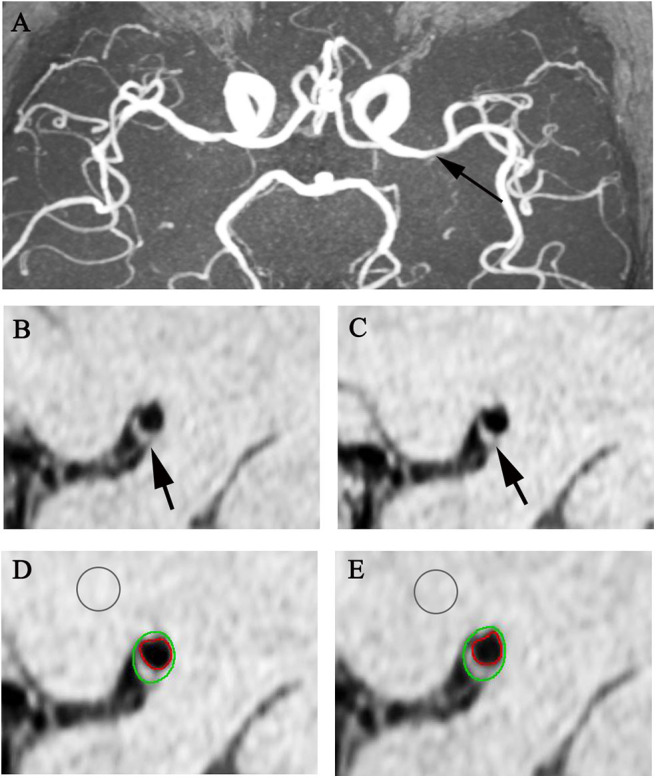
Representative MR images showing enhancement index measurement. A 65-year-old man with acute stroke showed severe left MCA stenosis on MRA (**A** arrow). After matching pre- **(B,D)** and post-contrast T1w images **(C,E)** at the most stenotic site, the plaque enhancement index of the plaque (arrows) is 2.82%.

Plaque enhancement was quantified by manually tracing the vessel lumen and the outer boundary of vessel wall at the most stenotic site of MCA and measuring the signal intensity of plaque (SI _plaque_) on pre- and matched post-contrast T1-weighted images. The signal intensity of gray matter (SI _graymatter_) was measured by manually drawing a round region of 10–12 mm^2^ at the adjacent normal gray matter on matched pre- and post-contrast T1-weighted images, respectively. The plaque enhancement index was calculated to characterize the extent of plaque contrast enhancement by using the following formula: Enhancement index = [(SI _plaque_/SI _graymatter_ on post-contrast T1w images) – (SI _plaque_/SI _graymatter_ on pre-contrast T1w images)] / (SI _plaque_/SI _graymatter_ on pre-contrast T1w images) ^*^ 100% (Figures 1, 2), according to the methods described in a previous study (26).

Lesions were classified as symptomatic or asymptomatic according to the brain infarct on DWI and/or signs of neurological deficits during the ischemic events. A plaque was considered as symptomatic if: (1) it is the only lesions within the ipsilateral MCA distribution of stroke, or (2) it is the most stenotic plaque when tandem lesions were present within the same vascular distribution of stoke. A plaque was defined as asymptomatic if: (1) it is not within the vascular distribution of stoke, or (2) it is within the vascular distribution of stoke but not the most stenotic lesion.

### Statistical Analysis

All data analyses were conducted using the SPSS 20.0 software package (SPSS, Inc., USA). Independent-samples *T*-test (for normally distributed variables) or Mann-Whitney *U*-test (for non-normally distributed variables) was used to compare means between symptomatic and asymptomatic groups. ANOVA and *post-hoc* pairwise comparisons (LSD) were used to compare means among acute, subacute and chronic groups. Chi-square or Fisher's exact test was used to compare categorical variables. Univariate and multivariate linear mixed regression analysis were performed to assess how the variables, such as stroke phases (acute, subacute, chronic), symptomatic status, plaque load, luminal stenosis, and IPH, were related to contrast enhancement indexes. A two-tailed *P* < 0.05 was considered indicative of a significant difference.

## Results

### Patient Characteristics

A total of 58 patients (median age: 64; male: 60%) were recruited in the present study. The clinical characteristics are as follows: hypertension (60%), hyperlipidemia (45%), diabetes (26%), smoking (36%) and previous stroke (21%) (Table 1). According to the time interval between symptoms onset and HRMRI, 34 patients were classified as acute phase (<4 weeks), 13 patients as subacute phase (4–12 weeks), and 11 patients as chronic phase (>12 weeks), with the mean interval 9 days, 39 days and 3 years and 3 months, respectively. No patients had a recurrent stroke or TIA from the onset of ischemic events to HRMRI scan.

**Table 1:** Demographic and Clinical Characteristics of Patients with Acute, Subacute, and Chronic Stroke.

**Characteristics**	**Acute (*n* = 34)**	**Subacute (*n* = 13)**	**Chronic (*n* = 11)**	***P***
Age	61 ± 13	65 ± 10	70 ± 5	0.07
Male	20 (59%)	8 (62%)	7 (64%)	0.957
Hypertension	20 (59%)	7 (54%)	8 (73%)	0.622
Hyperlipidemia	15 (44%)	5 (39%)	6 (55%)	0.73
Diabetes mellitus	9 (27%)	3 (23%)	3 (27%)	0.966
Smoking	11 (33%)	7 (54%)	3 (27%)	0.315
Old stroke	7 (21%)	2 (15%)	3 (27%)	0.777

### Plaque Enhancement and Symptomatic Status

A total of 89 MCA plaques were identified in 58 patients, including 53 (60%) symptomatic and 36 (40%) asymptomatic. Compared with asymptomatic plaques, symptomatic plaques had a higher contrast enhancement index (symptomatic vs. asymptomatic: 28.4 ± 22.4 vs. 16.7 ± 18.1, *p* = 0.011). Among all plaques, 57 (64%) lesions were in the acute phase, 18 (20%) in the subacute, and 14 (16%) in the chronic phase. When only plaques in the acute phase were included, an even more significant difference in the contrast enhancement index was observed between symptomatic and asymptomatic plaques (symptomatic vs. asymptomatic: 38.9 ± 18.2 vs. 18.2 ± 16.2, *p* < 0.001). Conversely, within the subacute and chronic plaques, there was no significant difference in the plaque enhancement strength (subacute: symptomatic vs. asymptomatic, 22.0 ± 22.8 vs. 17.5 ± 26.2, *p* = 0.700; chronic: symptomatic vs. asymptomatic, 5.0 ± 10.1 vs. 2.5 ± 10.4, *p* = 1.000).

### Plaque Enhancement and Stroke Phase

The strengths of plaque contrast enhancement represented by the enhancement index were compared among acute, subacute and chronic groups. There was a trend that enhancement indexes in subacute plaques decreased as compared with acute plaques, but the difference was not significant (acute vs. subacute: 29.5% ± 20.1 vs. 20.3% ± 23.5, *p* = 0.087). Chronic plaques revealed a significant decrease in enhancement indexes compared with both acute and subacute plaques (acute vs. chronic: 29.5% ± 20.1 vs. 4.4% ± 9.8, *p* < 0.001; subacute vs. chronic: 20.3% ± 23.5 vs. 4.4% ± 9.8, *p* = 0.026). No significant difference was found in plaque load (*p* = 0.193), luminal stenosis (*p* = 0.547), or IPH (*p* = 0.548) among acute, subacute, and chronic groups (Table 2).

**Table 2:** Comparison of Plaque Characteristics among Acute, Subacute, and Chronic Plaques.

		**Acute**	**Subacute**	**Chronic**	***p*-value**
Enhancement Indexes (%)	All	29.5 ± 20.1	20.3 ± 23.5	4.4 ± 9.8	<0.001^*^
	Symptomatic	38.9 ± 18.2	22.0 ± 22.8	5.0 ± 10.1	<0.001^*^
	Asymptomatic	18.2 ± 16.2	17.5 ± 26.2	2.5 ± 10.4	0.378
Plaque load (%)	All	81.0 ± 10.1	76.7 ± 17.7	75.5 ± 11.2	0.193
	Symptomatic	93.9 ± 8.4	77.7 ± 21.3	76.4 ± 11.9	0.162
	Asymptomatic	77.5 ± 10.9	75.2 ± 11.2	71.9 ± 9.0	0.647
Luminal stenosis (%)	All	59.7 ± 25.0	55.2 ± 26.6	52.3 ± 21.8	0.547
	Symptomatic	66.8 ± 24.3	62.9 ± 26.0	55.1 ± 21.8	0.393
	Asymptomatic	51.2 ± 23.5	43.1 ± 24.5	41.8 ± 22.1	0.627
IPH	All	4 (36.4%)	4 (36.4%)	3 (27.3%)	0.548
	Symptomatic	4 (44.4%)	2 (22.2%)	3 (33.3%)	0.472
	Asymptomatic	0 (0.0%)	2 (100.0%)	0 (0.0%)	0.071

*IPH, intraplaque hemorrhage*.

* *p < 0.05*.

**Figure 3:**
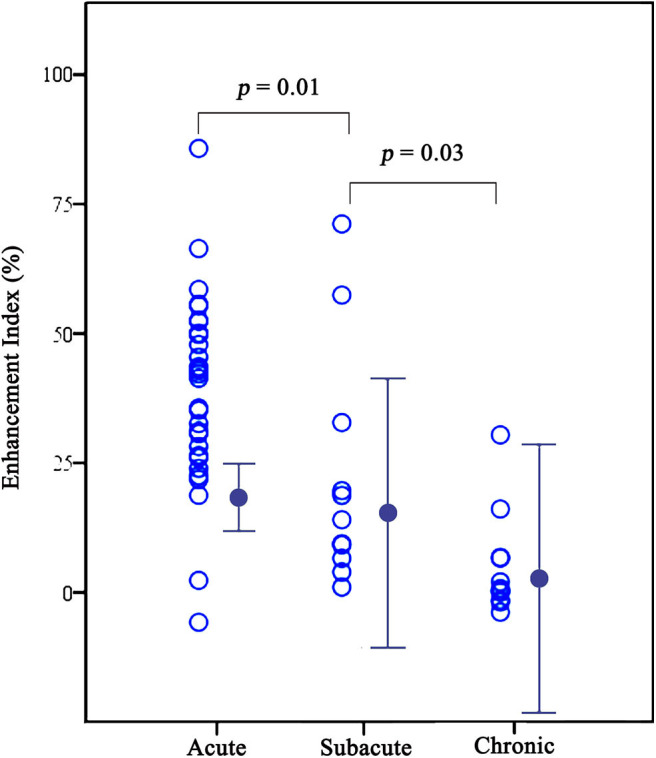
Graphs comparing enhancement indexes among acute, subacute, and chronic plaques for symptomatic lesions. The enhancement indexes decreased gradually after symptom onset (38.9 ± 18.2, 22.0 ± 22.8, and 5.0 ± 10.1 for acute, subacute and chronic phase, respectively, all *p* < 0.05).

Among the 53 symptomatic lesions, 31 (58%) were acute lesions, 11 (21%) were subacute lesions, and 11 (21%) were chronic lesions. Enhancement indexes decreased gradually after ischemic events (38.9% ± 18.2, 22.0% ± 22.8, and 5.0% ± 10.1 for acute, subacute and chronic phase, respectively, *p* < 0.001) (Table 2 and Figure 3). In pairwise comparisons, the enhancement strength of acute plaques was higher than subacute plaques (*p* = 0.01), and that of subacute plaques was considerably higher than chronic plaques (*p* = 0.03).

### Univariate and Multivariate Analysis

The univariate analysis showed that stroke phases (*B* = 23.853, *p* < 0.001 for acute vs. chronic; *B* = 15.434, *p* = 0.028 for subacute vs. chronic), symptomatic status (*B* = 10.275, *p* = 0.035), and luminal stenosis (*B* = 0.285, *p* = 0.002) were associated with contrast enhancement. Plaque load and IPH did not show significant relationships with plaque enhancement. The association of plaque enhancement with stroke phases (*B* = 27.554, *p* < 0.001 for acute vs. chronic; *B* = 17.442, *p* = 0.009 for subacute vs. chronic), symptomatic status (*B* = 11.545, *p* = 0.008), and area stenosis (*B* = 0.164, *p* = 0.050) remained strong in multivariate analysis after adjusted for age, sex and IPH (Table 3).

**Table 3:** Univariate and multivariate linear mixed regression model for independent associations with enhancement indexes.

	**Univariate regression**		**Multivariate regression^#^**	
	**Beta coefficient (95% CI)**	***P*-value**	**Beta coefficient (95% CI)**	***P*-value**
**Stroke phase**				
Acute	23.853 (12.308–35.399)	<0.001^*^	27.554 (15.648–39.460)	<0.001^*^
Subacute	15.434 (1.726–29.142)	0.028^*^	17.442 (4.397–30.486)	0.009^*^
Chronic	1	–	1	–
**Symptomatic status**	10.275 (0.735–19.816)	0.035^*^	11.545 (3.146–19.945)	0.008^*^
**Luminal stenosis**	0.285 (0.110–0.459)	0.002^*^	0.164 (0.001–0.329)	0.050^*^
**Plaque load**	-0.018 (-1.511–1.475)	0.981	–	–
**IPH**	0.336 (-6.892–19.973)	0.336	–	–

# *Adjusted for age, sex, and intraplaque hemorrhage (IPH)*.

*CI, Confidential Interval*.

* *p < 0.05*.

## Discussion

In this study, we quantitatively assessed the enhancement patterns of MCA by using contrast-enhanced HRMRI and revealed that both the symptomatic status and stroke phases were associated with the extent of plaque enhancement. Among acute plaques, plaque enhancement was strongly associated with the symptomatic ischemic presentation. Among symptomatic plaques, enhancement indexes decreased gradually after the onset of ischemic events. These findings may provide insight into stroke risk stratification.

Substantial contrast enhancement within the carotid plaque has been shown to be correlated with extensive neovascularization and inflammatory cell infiltration in histological studies (14, 27), and be associated with elevated serum markers of inflammation, such as interleukin-6 (28). Given the fundamental role of inflammation in the progression and rupture of atherosclerotic plaque, plaque enhancement identified on HRMRI seems to be a vital indicator of plaque vulnerability. Several extracranial artery atherosclerosis studies demonstrated that plaque enhancement was closely related to recent cerebrovascular ischemic events (29, 30), suggesting the potential of plaque enhancement as an MRI marker for identifying high-risk atherosclerotic plaque.

In the current study, plaque enhancement in the acute lesions was stronger in symptomatic MCA plaques than that in asymptomatic plaques, consistent with the recent findings showing that the intracranial atherosclerotic plaque tended to be more frequently enhanced in the arteries ipsilateral to the infarcts (31–34). This indicates that plaque enhancement in intracranial lesions may also serve as a marker of plaque instability, although the pathological validation of the intracranial plaques is generally unattainable. However, while the strong association between plaque enhancement and ischemic events was observed in acute lesions, the chronic plaques, either symptomatic or asymptomatic, rarely showed significant plaque enhancement.

A previous study of coronary artery atherosclerosis has demonstrated a significant regression in plaque enhancement during the 3-month post-infarction period. The decrease was paralleled by declines in C-reactive protein, indicating that the contrast-enhanced cardiac magnetic resonance offers the potential to indirectly visualize the transient inflammatory process in coronary artery atherosclerosis by characterizing the changes in plaque enhancement (35). In this study, we also observed a trend toward a decrease in plaque enhancement strength within symptomatic MCA atherosclerosis after the onset of ischemic events, which may further support the potential link between altered plaque enhancement and dynamic inflammatory activity. Limited studies have investigated the changes of plaque enhancement after the onset of ischemic events. A prior MRI study evaluated the presence and strength of enhancement in symptomatic intracranial lesions, demonstrating that all acute plaques showed substantial enhancement while a majority of chronic plaques had mild or no enhancement (22). Another 7 Tesla MRI study followed up stroke or TIA patients in 1 month after symptom onset, in which only 17% of atherosclerotic lesions showed changes in the presence of plaque enhancement or enhancement patterns over time (36). However, 1 month is relatively short as compared to the long natural course of inflammatory activity. In a small study of 7 stroke or TIA patients with symptomatic >70% of stenosis in the M1 segment, Abe et al. observed an enhancement decrease over a 7-month follow-up period in 4 of 7 patients (23), implying that the enhancement patterns may change after a longer period of stroke onset. The mean period of chronic stroke patients in our study was 3 years and 3 months, which may explain why we observed a considerable low enhancement index in chronic lesions. Of note, in Abe's study, two of the three patients who did not show enhancement reduction had a recurrent stroke during the follow-up (23). Plaque enhancement is considered as an imaging marker of future stroke risk (37), we thus speculate that the less enhanced pattern of chronic lesions in our study may be attributable to no stroke recurrence in chronic stroke patients. Additionally, while acute and subacute patients received mono antiplatelet treatment, the chronic group was given dual antiplatelet treatment for 4 weeks before switching to monotherapy, which may also contribute to the less significant plaque enhancement pattern in the chronic group.

The mechanisms underlying plaque enhancement is complex and unclear. Some other plaque features or components could also have confounding effects on the association of plaque enhancement with either stroke phase or symptomatic status. Consistent with a prior study (33), we found that degree of luminal stenosis was closely associated with plaque enhancement, implying that the decreased enhancement in chronic lesions may also be explained by plaque volume regression. However, no statistically significant difference in plaque load or area stenosis was found among the acute, subacute and chronic groups, suggesting that luminal stenosis may not account for the plaque regression from the acute to chronic phase in our study. IPH is attributed to inherently immature, fragile neovessels that surround and invade the plaque, and could also contribute to the entry of gadolinium into the plaque (38). However, no association between IPH and plaque enhancement was found in this study.

There are some limitations in our study. First, this is a cross-sectional study comparing the strength of plaque enhancement in stroke patients of acute, subacute, and chronic phases, respectively, and follow-ups at various time points were not performed to evaluate the time-course changes in plaque enhancement. The enhancement differences in distinct groups could be due to the selection bias. However, the chronic patients in our study were consecutively recruited, and all the MCA plaque showed a considerably low enhancement index compared to acute patients, implying that plaque enhancement cannot persist for a long period after stroke onset. Second, although the relationship of contrast enhancement with inflammation has been well-established within the extracranial plaques, the exact mechanism accounting for intracranial plaque enhancement remains largely unexplored. Further studies by using molecular contrast agents to target specific inflammatory markers may assist in clarifying the basis of enhancement (39).

In summary, the quantitative assessment of MCA plaque shows an association between plaque enhancement and ischemic stroke, and also reveals temporal regression of plaque enhancement relative to the onset of the ischemic events. If the uptake of contrast in ICAD has the same mechanisms as that in extracranial and coronary vessels, intracranial plaque enhancement may act as a promising imaging biomarker for plaque activity.

## Data Availability Statement

The datasets generated for this study are available on request to the corresponding author.

## Ethics Statement

The studies involving human participants were reviewed and approved by Institutional Review Board of the Chinese University of Hong Kong. The patients/participants provided their written informed consent to participate in this study.

## Author Contributions

W-JY recruited patients, analyzed MRI data, and drafted the manuscript. JA acquired MRI data and helped to draft the manuscript. YS participated in study coordination and recruited patients. SW acquired the MRI data. K-SW and TL participated in the design and coordination of the study. WC and X-YC conceived the study, participated in its design and coordination, and revised the manuscript. All authors contributed to the article and approved the submitted version.

## Conflict of Interest

The authors declare that the research was conducted in the absence of any commercial or financial relationships that could be construed as a potential conflict of interest.

## References

[1] Wang Y Zhao X Liu L Soo YO Pu Y Pan Y. Prevalence and outcomes of symptomatic intracranial large artery stenoses and occlusions in China: the Chinese intracranial atherosclerosis (CICAS) study. Stroke. (2014) 45:663–9. 10.1161/STROKEAHA.113.003508 24481975

[2] Holmstedt CA Turan TN Chimowitz MI. Atherosclerotic intracranial arterial stenosis: risk factors, diagnosis, and treatment. Lancet Neurol. (2013) 12:1106–14. 10.1016/S1474-4422(13)70195-9 24135208 PMC4005874

[3] Marnane M Prendeville S McDonnell C Noone I Barry M Crowe M. Plaque inflammation and unstable morphology are associated with early stroke recurrence in symptomatic carotid stenosis. Stroke. (2014) 45:801–6. 10.1161/STROKEAHA.113.003657 24481971

[4] Chen XY Wong KS Lam WW Zhao HL Ng HK. Middle cerebral artery atherosclerosis: histological comparison between plaques associated with and not associated with infarct in a postmortem study. Cerebrovasc Dis. (2008) 25:74–80. 10.1159/000111525 18033961

[5] Yuan C Mitsumori LM Beach KW Maravilla KR. Carotid atherosclerotic plaque: noninvasive MR characterization and identification of vulnerable lesions. Radiology. (2001) 221:285–99. 10.1148/radiol.2212001612 11687667

[6] Saam T Hatsukami TS Takaya N Chu B Underhill H Kerwin WS The vulnerable, or high-risk, atherosclerotic plaque: noninvasive MR imaging for characterization and assessment. Radiology. (2007) 244:64–77. 10.1148/radiol.2441051769 17581895

[7] Xu WH Li ML Gao S Ni J Zhou LX Yao M. Plaque distribution of stenotic middle cerebral artery and its clinical relevance. Stroke. (2011) 42:2957–9. 10.1161/STROKEAHA.111.618132 21799160

[8] Qiao Y Guallar E Suri FK Liu L Zhang Y Anwar Z. MR imaging measures of intracranial atherosclerosis in a population-based study. Radiology. (2016) 280:860–8. 10.1148/radiol.2016151124 27022858 PMC5006718

[9] Teng Z Peng W Zhan Q Zhang X Liu Q Chen S. An assessment on the incremental value of high-resolution magnetic resonance imaging to identify culprit plaques in atherosclerotic disease of the middle cerebral artery. Eur Radiol. (2016) 26:2206–14. 10.1007/s00330-015-4008-5 26376883 PMC4902836

[10] Xu WH Li ML Gao S Ni J Yao M Zhou LX. Middle cerebral artery intraplaque hemorrhage: prevalence and clinical relevance. Ann Neurol. (2012) 71:195–8. 10.1002/ana.22626 22367991

[11] Dieleman N Yang W Abrigo JM Chu WC van der Kolk AG Siero JC Magnetic resonance imaging of plaque morphology, burden, and distribution in patients with symptomatic middle cerebral artery stenosis. Stroke. (2016) 47:1797–802. 10.1161/STROKEAHA.116.013007 27301944 PMC4927221

[12] Zhang S Cai J Luo Y Han C Polissar NL Hatsukami TS. Measurement of carotid wall volume and maximum area with contrast-enhanced 3D MR imaging: initial observations. Radiology. (2003) 228:200–5. 10.1148/radiol.2281020484 12832583

[13] Wasserman BA Smith WI Trout HH 3rd Cannon RO 3rd Balaban RS Arai AE. Carotid artery atherosclerosis: *in vivo* morphologic characterization with gadolinium-enhanced double-oblique MR imaging initial results. Radiology. (2002) 223:566–73. 10.1148/radiol.2232010659 11997569

[14] Millon A Boussel L Brevet M Mathevet JL Canet-Soulas E Mory C. Clinical and histological significance of gadolinium enhancement in carotid atherosclerotic plaque. Stroke. (2012) 43:3023–8. 10.1161/STROKEAHA.112.662692 22923447

[15] Wasserman BA. Advanced contrast-enhanced MRI for looking beyond the lumen to predict stroke: building a risk profile for carotid plaque. Stroke. (2010) 41(Suppl. 10): S12–6. 10.1161/STROKEAHA.110.596288 20876485

[16] Kerwin WS O'Brien KD Ferguson MS Polissar N Hatsukami TS Yuan C. Inflammation in carotid atherosclerotic plaque: a dynamic contrast-enhanced MR imaging study. Radiology. (2006) 241:459–68. 10.1148/radiol.2412051336 16966482 PMC1820770

[17] Gupta A Baradaran H Al-Dasuqi K Knight-Greenfield A Giambrone AE Delgado D. Gadolinium enhancement in intracranial atherosclerotic plaque and ischemic stroke: a systematic review and meta-analysis. J Am Heart Assoc. (2016) 5:e003816. 10.1161/JAHA.116.003816 27528408 PMC5015301

[18] Wang W Yang Q Li D Fan Z Bi X Du X. Incremental value of plaque enhancement in patients with moderate or severe basilar artery stenosis: 3.0 t high-resolution magnetic resonance study. Biomed Res Int. (2017) 2017:4281629. 10.1155/2017/4281629 29075645 PMC5623789

[19] Harteveld AA van der Kolk AG van der Worp HB Dieleman N Zwanenburg JJM Luijten PR. Detecting intracranial vessel wall lesions with 7t-magnetic resonance imaging: patients with posterior circulation ischemia versus healthy controls. Stroke. (2017) 48:2601–4. 10.1161/STROKEAHA.117.017868 28701579

[20] de Havenon A Mossa-Basha M Shah L Kim SE Park M Parker D. High-resolution vessel wall MRI for the evaluation of intracranial atherosclerotic disease. Neuroradiology. (2017) 59:1193–202. 10.1007/s00234-017-1925-9 28942481

[21] Yang WJ Wong KS Chen XY. Intracranial atherosclerosis: from microscopy to high-resolution magnetic resonance imaging. J Stroke. (2017) 19:249–60. 10.5853/jos.2016.01956 28877564 PMC5647638

[22] Skarpathiotakis M Mandell DM Swartz RH Tomlinson G Mikulis DJ. Intracranial atherosclerotic plaque enhancement in patients with ischemic stroke. Am J Neuroradiol. (2013) 34:299–304. 10.3174/ajnr.A3209 22859280 PMC7965103

[23] Abe A Sekine T Sakamoto Y Harada-Abe M Takagi R Suda S. Contrast-Enhanced high-resolution mri for evaluating time course changes in middle cerebral artery plaques. J Nippon Med Sch. (2018) 85:28–33. 10.1272/jnms.2018_85-4 29540643

[24] Leung TW Wang L Soo YO Ip VH Chan AY Au LW. Evolution of intracranial atherosclerotic disease under modern medical therapy. Ann Neurol. (2015) 77:3. 10.1002/ana.24340 25557926

[25] Kernan WN Ovbiagele B Black HR Bravata DM Chimowitz MI Ezekowitz MD. Guidelines for the prevention of stroke in patients with stroke and transient ischemic attack: a guideline for healthcare professionals from the american heart association/American stroke association. Stroke. (2014) 45:2160–236. 10.1161/STR.0000000000000024 24788967

[26] Lou X Ma N Ma L Jiang WJ. Contrast-enhanced 3T high-resolution MR imaging in symptomatic atherosclerotic basilar artery stenosis. Am J Neuroradiol. (2013) 34:513–7. 10.3174/ajnr.A3241 22878005 PMC7964910

[27] Yuan C Kerwin WS Ferguson MS Polissar N Zhang S Cai J. Contrast-enhanced high resolution MRI for atherosclerotic carotid artery tissue characterization. J Magn Reson Imaging. (2002) 15:62–7. 10.1002/jmri.10030 11793458

[28] Weiss CR Arai AE Bui MN Agyeman KO Waclawiw MA Balaban RS. Arterial wall MRI characteristics are associated with elevated serum markers of inflammation in humans. J Magn Reson Imaging. (2001) 14:698–704. 10.1002/jmri.10023 11747026

[29] Qiao Y Etesami M Astor BC Zeiler SR Trout HH 3rd Wasserman BA. Carotid plaque neovascularization and hemorrhage detected by MR imaging are associated with recent cerebrovascular ischemic events. AJNR Am J Neuroradiol. (2012) 33:755–60. 10.3174/ajnr.A2863 22194363 PMC3979429

[30] Kerwin WS Oikawa M Yuan C Jarvik GP Hatsukami TS. MR imaging of adventitial vasa vasorum in carotid atherosclerosis. Magn Reson Med. (2008) 59:507–14. 10.1002/mrm.21532 18306402

[31] Vakil P Vranic J Hurley MC Bernstein RA Korutz AW Habib A. T1 gadolinium enhancement of intracranial atherosclerotic plaques associated with symptomatic ischemic presentations. AJNR Am J Neuroradiol. (2013) 34:2252–8. 10.3174/ajnr.A3606 23828109 PMC7965223

[32] Qiao Y Zeiler SR Mirbagheri S Leigh R Urrutia V Wityk R. Intracranial plaque enhancement in patients with cerebrovascular events on high-spatial-resolution MR images. Radiology. (2014) 271:534–42. 10.1148/radiol.13122812 24475850 PMC4263625

[33] Ryu CW Jahng GH Shin HS. Gadolinium enhancement of atherosclerotic plaque in the middle cerebral artery: relation to symptoms and degree of stenosis. AJNR Am J Neuroradiol. (2014) 35:2306–10. 10.3174/ajnr.A4038 25012673 PMC7965297

[34] Wu F Ma Q Song H Guo X Diniz MA Song SS. Differential features of culprit intracranial atherosclerotic lesions: a whole-brain vessel wall imaging study in patients with acute ischemic stroke. J Am Heart Assoc. (2018) 7:e009705. 10.1161/JAHA.118.009705 30033434 PMC6201468

[35] Ibrahim T Makowski MR Jankauskas A Maintz D Karch M Schachoff S. Serial contrast-enhanced cardiac magnetic resonance imaging demonstrates regression of hyperenhancement within the coronary artery wall in patients after acute myocardial infarction. JACC Cardiovasc Imaging. (2009) 2:580–8. 10.1016/j.jcmg.2008.12.029 19442944

[36] van der Kolk AG Zwanenburg JJ Brundel M Biessels GJ Visser F Luijten PR. Distribution and natural course of intracranial vessel wall lesions in patients with ischemic stroke or TIA at 7.0 Tesla MRI. Eur Radiol. (2015) 25:6. 10.1007/s00330-014-3564-4 25577517

[37] Kim JM Jung KH Sohn CH Moon J Shin JH Park J. Intracranial plaque enhancement from high resolution vessel wall magnetic resonance imaging predicts stroke recurrence. Int J Stroke. (2016) 11:1692–700. 10.1177/1747493015609775 26783308

[38] Virmani R Kolodgie FD Burke AP Finn AV Gold HK Tulenko TN. Atherosclerotic plaque progression and vulnerability to rupture: angiogenesis as a source of intraplaque hemorrhage. Arterioscler Thromb Vasc Biol. (2005) 25:2054–61. 10.1161/01.ATV.0000178991.71605.18 16037567

[39] Nahrendorf M Jaffer FA Kelly KA Sosnovik DE Aikawa E Libby P. Noninvasive vascular cell adhesion molecule-1 imaging identifies inflammatory activation of cells in atherosclerosis. Circulation. (2006) 114:1504–11. 10.1161/CIRCULATIONAHA.106.646380 17000904

